# Petroleum extract of *Farfarae Flos* alleviates nasal symptoms by regulating the Th1-Th2 cytokine balance in a mouse model of Allergic Rhinitis

**DOI:** 10.7150/ijms.52915

**Published:** 2021-01-01

**Authors:** Yongyan Wu, Xiaojuan Zhao, Jiajia Cui, Yujia Guo, Xiwang Zheng, Yuliang Zhang, Min Niu, Zhenyu Li, Shuxin Wen, Wei Gao

**Affiliations:** 1 Shanxi Key Laboratory of Otorhinolaryngology Head and Neck Cancer, First Hospital of Shanxi Medical University, Taiyuan 030001, Shanxi, China.; 2 Shanxi Province Clinical Medical Research Center for Precision Medicine of Head and Neck Cancer, First Hospital of Shanxi Medical University, Taiyuan 030001, Shanxi, China.; 3 Department of Otolaryngology Head & Neck Surgery, First Hospital of Shanxi Medical University, Taiyuan 030001, Shanxi, China.; 4 Key Laboratory of Cellular Physiology, Ministry of Education, Shanxi Medical University, Taiyuan 030001, Shanxi, China.; 5 Modern Research Center for Traditional Chinese Medicine, Shanxi University, Taiyuan 030006, Shanxi, China.; 6 Department of Otolaryngology Head & Neck Surgery, Shanxi Bethune Hospital, Taiyuan 030032, Shanxi, China.

**Keywords:** Allergic Rhinitis, *Farfarae Flos*, IgE, Th1-Th2, Cytokine

## Abstract

*Farfarae Flos* is a traditional Chinese medicine that has long been used to treat allergies. In this study, we aimed to investigate the effect of a petroleum extract of *Farfarae Flos* (PEFF) in a mouse model of allergic rhinitis (AR) and to explore the underlying molecular mechanisms of action. An animal model of AR was established by sensitization and challenge of BALB/c mice with ovalbumin (OVA). PEFF was administered intranasally and AR nasal symptoms were assessed on a semi-quantitative scale according to the frequencies of nose rubbing and sneezing and the degree of rhinorrhea. The mechanism of action of PEFF was evaluated by histological analysis of nasal mucosa architecture and inflammatory status; ELISA-based quantification of serum OVA-specific IgE, interferon-γ (IFN-γ), and interleukin-4 (IL-4) concentrations; and immunohistochemical and western blot analysis of T-bet and GATA3 protein expression in nasal mucosa and spleen tissues. The results showed intranasal administration of PEFF alleviated AR symptom scores and reduced both the infiltration of inflammatory cells and tissue damage in the nasal mucosa. PEFF significantly decreased serum concentrations of OVA-specific IgE (*P*<0.01) and IL-4 (*P*<0.05) and significantly increased IFN-γ (*P*<0.01). PEFF also upregulated the expression of T-bet protein (*P*<0.05) but downregulated GATA3 protein (*P*<0.05) in nasal mucosa and spleen tissues. In conclusion, PEFF effectively reduces AR nasal symptoms and serum IgE levels in a mouse model and may act by correcting the imbalance between Th1 and Th2 responses.

## Introduction

Allergic rhinitis (AR) is a non-infectious chronic inflammatory disease of the nasal mucosa that is mainly mediated by specific IgE induced by exposure to allergens 1. Typical symptoms of AR include paroxysmal sneezing, rhinorrhea, nasal congestion, and nasal itching. Epidemiological studies report that AR affects 10% to 40% of the global population and 4% to 38% of the Chinese population 2, 3. As a chronic inflammatory disease of the respiratory tract, AR has a severe impact on quality of life and is a heavy socioeconomic burden 4, 5. Furthermore, AR is a risk factor for the development of asthma. Glucocorticoids, antihistamines, leukotriene antagonists, mast cell stabilizers, nasal decongestants, and anticholinergics are commonly used for the treatment of AR 6. However, although these medicines can rapidly relieve AR symptoms, they can also cause serious adverse reactions. For example, long-term use of nasal glucocorticoids causes nasal dryness and bleeding and throat irritation 7. Antihistamines can relieve many nasal symptoms, such as itching, sneezing and runny nose, they have no effect on airway obstruction 8. Similarly, although allergen-specific immunotherapy is an effective method of changing the natural course of the disease through immunoregulatory mechanisms, its efficacy is considerably influenced by factors such as patient compliance, allergen type, and systemic adverse reactions. Allergen-specific immunotherapy is also expensive, which has greatly restricted its promotion and use to treat AR in China 9. Thus, there is a great need for the development of safer and lower-cost therapeutic agents for AR.

Traditional Chinese medicine has been used as a treatment for AR for hundreds of years, and it has several advantages over many western medicines, including lower side effects, stable curative effects, and mechanism-based regulation of the pathogenesis of allergies 10, 11.* Farfarae Flos* (FF) is the dried bud of the* Tussilago farfara L.* plant, which is considered “the best way to cure cough” in “Shen Nong's Herbal Classic” 12. FF has been used in many Chinese medicines for the treatment of chronic cough, asthma, bronchitis, and tuberculosis. The chemical composition of FF has been defined and includes sesquiterpenoids, triterpenes, flavonoids, and alkaloids with anti-inflammatory 13, anti-cancer 14, neuroprotective 15, and anti-viral 16 biological activities. Lee et al. reported that tussilagonone isolated from FF suppresses the production of inflammatory mediators such as nitric oxide and prostaglandin E2 17. A recent study also suggested that FF extracts inhibit leukocytosis in ammonia-induced acute airway inflammation 18.

AR is an immunological disorder with many underlying causes, including environmental and genetic contributions. However, it is believed to result from an imbalance between Th1 and Th2 immune responses, with a dominant Th2 element 19. Li et al. showed that a petroleum extract of FF (PEFF) is therapeutic in an ovalbumin (OVA)-induced rat model of asthma, which may be related to rebalancing of Th1-Th2 cell responses *via* the nuclear factor erythroid-2-related actor 2/heme oxygenase 1 pathway 20. Therefore, we speculated that PEFF may also have therapeutic benefits in AR.

In the present study, we tested this hypothesis by establishing a mouse model of AR induced by OVA sensitization followed by local challenge. We analyzed the effects of intranasal (i.n.) administration of PEFF on AR symptoms and mediators, and investigated its underlying biological mechanisms. Our findings shed light on the molecular mechanisms of action of PEFF and suggest that it may have clinical utility for the prevention and treatment of AR.

## Methods

### Reagents

OVA, aluminum hydroxide, and DMSO were purchased from Sigma-Aldrich (St. Louis, MO, USA). IgE ELISA Ready-Set-Go kits were obtained from Sangon Biotech (Shanghai, China), and IFN-γ and IL-4 ELISA kits were obtained from Arigo Biolaboratories Corporation (Shanghai, China). Budesonide (BUD) was obtained from AstraZeneca (Taiyuan, China). The anti-GATA-3 antibody was obtained from Proteintech (Wuhan, China), and anti-T-box expressed in T cells (T-bet) antibody was purchased from Cell Signaling Technology (Danvers, MA, USA).

### Preparation, major components detection, and dissolution of PEFF

*Farfarae Flos* (FF) was obtained from a local drug store and authenticated as the buds of *Tussilago farfara L.* by Prof. Zhenyu Li. Voucher specimens (No. KD-34) were deposited in the herbarium of the Modern Research Center for Traditional Chinese Medicine of Shanxi University. The preparation and chemical characterization of PEFF have previously been reported by us 20. In brief, the dried FF flower buds (3 kg) were ground to a powder and extracted three times for 60 min each with 4 L of petroleum ether (60-90℃) with ultrasonication. The combined extract was then concentrated under reduced pressure (yield 6.5 g of PEFF) and used for experiments.

In previous studies 20, the typical 1H NMR spectrum of PEFF was obtained. In the region of δ 4.5-6.5 and δ 0.8-1.2, the signals corresponding to the doubles bonds and methyl groups of the sesquiterpenoids were obviously detected. In addition, seven compounds were identified in PEFF by LC-MS analysis. Finally, we proved that the sesquiterpenoids were present as the major components in the PEFF.

The sesquiterpenoids are soluble in lipophilic organic solvents. Considering the low toxicity and excellent permeability of the organic solvent dimethylsulfoxide (DMSO), the lyophilized PEFF was dissolved using 20% DMSO/saline. Furthermore, based on the PEFF treatment concentration in OVA-induced rat asthma models 20, low dose (LD, 0.01 g/kg), medium dose (MD, 0.02 g/kg), and high dose (HD, 0.04 g/kg) of PEFF were used.

### Animal experiments

All animal experiments were performed according to the Health Guide for the Care and Use of Laboratory Animals and were approved by the medical ethics committee of Shanxi Medical University. A total of 60 specific pathogen-free female BALB/c mice, aged 6-8 weeks, were purchased from Beijing Vital River Laboratory Animal Technology. They housed in ventilated isolation cages in a climate-controlled room (20 ± 1°C and 50-70% humidity) with a 12 h light-dark cycle. Animals were acclimated for one week before use to monitor for abnormal behavior.

The 60 mice were randomly divided into seven groups: (i) a control group (C, n = 8) was mock-sensitized and mock-challenged with saline; (ii-iii) two identically treated model groups (M1 and M2, n = 8/group) were sensitized and challenged with OVA and mock-treated with saline; (iv-vi) three PEFF treatment groups (n = 9/group) were sensitized, challenged with OVA, and treated by i.n. instillation of PEFF at a low dose (LD, 0.01 g/kg), medium dose (MD, 0.02 g/kg), or high dose (HD, 0.04 g/kg); and (vii) a positive control group (n = 9) was sensitized, challenged with OVA, and treated by i.n. instillation of BUD (P, 0.052 mg/kg).

**Figure 1:**
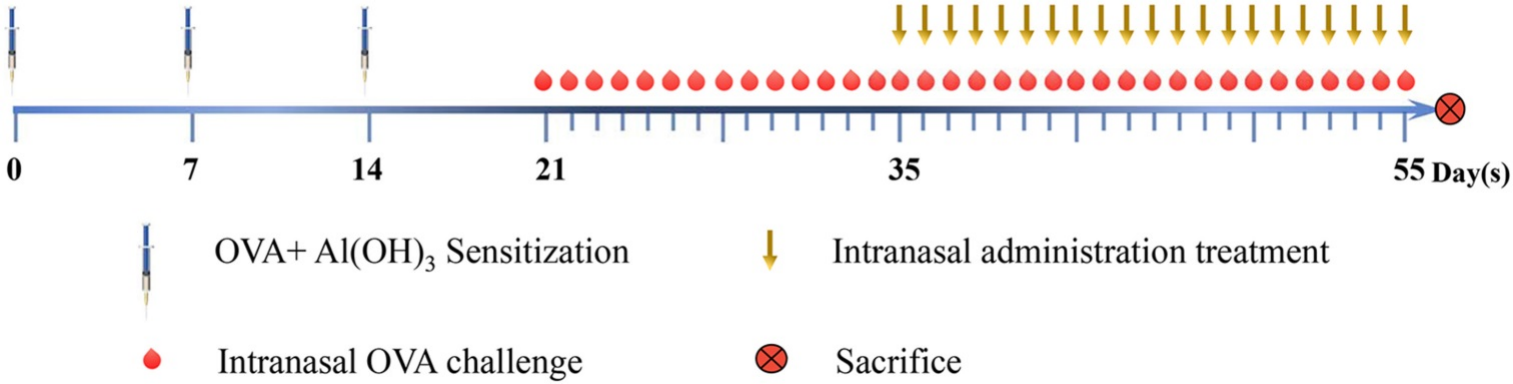
** Mouse model of AR.** Groups of BALB/c mice were sensitized by intraperitoneal injection of OVA and aluminum hydroxide on days 0, 7, and 14, followed by intranasal challenge with OVA once daily from day 21 to day 34. Mice in the model and treatment groups were administered 20% DMSO/saline and PEFF or BUD, respectively, once daily from day 35 to day 55 by intranasal instillation. Every other day between days 35 and 55, the mice also received OVA intranasally 1 h before treatment to maintain AR. Mice in the control group were administered saline intraperitoneally or intranasally on the same schedule. All mice were sacrificed on day 56.

Establishment of the AR model and timing of the treatments are described in Fig. 1. Briefly, the mice were sensitized by intraperitoneal (i.p.) injection of 200 μL saline containing 50 μg OVA and 2 mg aluminum hydroxide on days 0, 7, and 14. The mice were challenged i.n. with OVA (10 μL saline containing 100 μg/mL OVA) once daily from day 21 to day 34. The lyophilized PEFF was dissolved to the final concentration instilled into the nasal mucosa directly using 20% DMSO/saline. Between day 35 and day 55, the treatment groups were administered 20% DMSO/saline (M2), LD, MD, or HD PEFF, or BUD i.n. (10 μL per nostril) once daily, 1 h before the OVA challenge. The control mice received saline i.p. or i.n. on the same schedule. During the treatment period (days 35 to 55), the model (M2), PEFF, and BUD groups were challenged i.n. with 10 μL OVA (100 μg/mL) every other day to maintain the response. The data obtained from group M1 were used for evaluating the AR model, and data obtained from group M2 were used for evaluating the therapeutic effects of PEFF on AR.

### AR symptom score

As previously described 21, the nasal symptom score was based on three features, each graded on a four-point scale score between 0 and 3: (i) sneezing: 0, none; 1, 1-3 sneezes per 20 min; 2, 4-10 sneezes per 20 min; 3, ≥11 sneezes per 20 min; (ii) nasal mucus: 0, none; 1, mucus in nostrils; 2, mucus outflow from nostrils; 3, mucus outflow to face; and (iii) nasal itching: 0, none; 1, nose rubbing 1-2 times per min; 2, nose rubbing 3-5 times per min; 3, nose rubbing >6 times per min. The three scores were added to give a total score between 0 and 9 points. Animals recording a total score of ≥5 points were considered to have AR (Table 1). The symptom scores were measured every morning after the OVA challenge from day 21 to 55, and the observation time for symptom scoring is 20 minutes from the moment of OVA challenge.

### Histologic experiments

The tissue was anesthetized with 0.6% pentobarbital sodium after the successful modeling and the end of the treatment, blood was collected from the retro-orbital sinus, and the mice were euthanized. Nasal tissue was separated from the skin and muscles, fixed in 4% neutral buffered formalin, and decalcified for 14 days in 10% EDTA. The tissue was then embedded in paraffin, sliced into 3-μm-thick sections, and stained with hematoxylin and eosin (H&E). The sections were reviewed by a pathologist to assess the tissues.

### ELISA

Blood samples were allowed to stand for 30 min at room temperature and then centrifuged at 3000 × g for 15 min at room temperature. Serum was removed and the concentrations of OVA‑specific IgE, IFN-γ, and IL-4 were evaluated using the appropriate ELISA kits.

### Immunohistochemistry (IHC)

Prepared sections of nasal tissue were deparaffinized and antigen was retrieved by incubation in citrate buffer (pH 7.4) under high heat and pressure. Endogenous peroxidase activity was blocked by incubation of sections with 3% hydrogen peroxide in phosphate buffer solution (PBS) and non-specific staining was blocked with normal goat serum for 40 min at room temperature. The tissue sections were incubated overnight at 4°C in a humidified chamber with anti-T-bet (1:1600) or anti-GATA3 (1:500) antibodies diluted in PBS. After washing, the sections were incubated with biotin-conjugated goat anti-rabbit or anti-mouse IgG secondary antibodies for 30 min at room temperature. Antibody binding was visualized using a 3,3′-diaminobenzidine detection system. Slides were digitized using a Panoramic SCAN digital slide scanner with a Zeiss plan-apochromat objective (magnification 20×, aperture 0.8) and a Hitachi (HV-F22CL) 3CCD progressive scan color camera (resolution 0.2325 μm/pixel).

### Assessment of IHC staining

A semi-quantitative method was employed to assess T-bet and GATA3 protein staining in nasal mucosa tissue sections using Image-Pro plus 6.0 software. Five fields of view were randomly selected from each slice, and the intensity of positive staining within each field was calculated. The results are presented as the mean optical density (MOD) per μm^2^ in the five fields 22, 23.

### Western blot analysis

Total protein was isolated from tissues using a Mammalian Protein Extraction Kit (Biotech, Beijing, China) and protein concentrations were determined using a Bicinchoninic Acid protein assay kit (Yeasen Biotechnology). Proteins were separated by 10% sodium dodecyl sulfate-polyacrylamide gel electrophoresis and transferred to 0.2 μm PVDF membranes (Merck Millipore). The membranes were blocked with 10% nonfat milk and incubated overnight at 4°C with anti-T-bet and anti-GATA3 primary antibodies. The membranes were then washed and incubated with horseradish peroxidase-conjugated rabbit anti-goat IgG (H+L) (Beyotime), respectively, for 2 h at room temperature. The membranes were washed again and antibody binding was detected with ECL reagent (Advansta). Blots were also incubated with anti-glyceraldehyde 3-phosphate dehydrogenase (GAPDH) antibody (ProteinFind) as a loading control. All experiments were performed in triplicate.

### Statistical analysis

Data are presented as the mean ± standard deviation (SD). Differences between groups were determined using two-tailed Student's t test or ANOVA with SPSS 22.0 software (Chicago, IL, USA). A two-tailed P value of <0.05 was considered significant.

## Results

### Evaluation of an AR model in BALB/c mice

**Figure 2:**
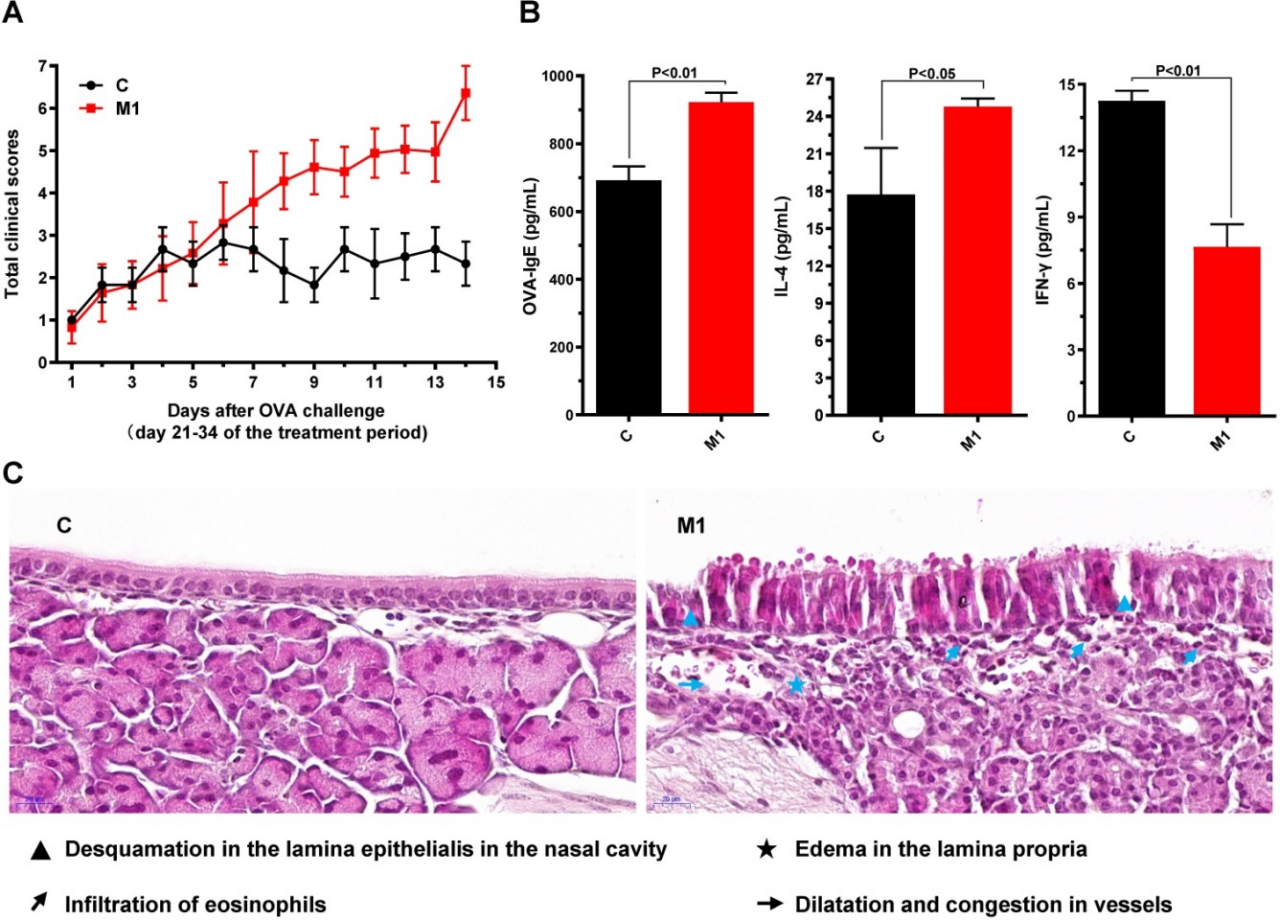
** Evaluation of AR in mice.** (**A**) Nasal symptom scores were calculated after observation of mice for 20 minutes after OVA challenge. (**B**) Serum OVA-specific IgE, IL-4, and IFN-γ concentrations were measured by ELISA. (**C**) The morphology and histology of nasal mucosa were evaluated by H&E staining of tissue sections (×400). Differences between groups were determined using two-tailed Student's *t* test. Data are presented as the mean ± SD. C, control group; M1, model group.

Groups of mice were sensitized on days 0, 7, and 14 and then challenged by i.n. administration of OVA once daily starting on day 21. The AR symptoms were scored according to the frequency of sneezing events and nose rubbing and the degree of rhinorrhea (maximum total score = 9, Table 1). Compared with the control group (C), the model group (M1) had a total nasal symptom score of ≥5, which was considered to reflect AR (Fig. 2A).

Serum levels of OVA-specific IgE, IFN-γ, IL-4 on day 55 were measured by ELISA. Compared with the control group, the model group had significantly higher levels of OVA-specific IgE (P<0.01) and IL-4 (P<0.05) but significantly lower levels of IFN-γ (P<0.01) (Fig. 2B).

Histopathological examination of the nasal mucosa of the control mice on day 34 revealed a normal architecture, pseudostratified ciliated columnar epithelium, uniformly arranged cilia, no detachment, and no obvious eosinophilic infiltration in the lamina propria. However, the nasal mucosa of the model group exhibited prominent alterations, with ciliary desquamation in the nasal epithelium, severe edema, angiectasis in the lamina propria, glandular organ hypertrophy, severe eosinophilic infiltration, and basement membrane thickening (Fig. 2C).

Collectively, these results indicate that the AR model had been successfully established in BALB/C mice.

### PEFF effects on nasal symptoms and nasal mucosa histology in mice with AR

**Figure 3:**
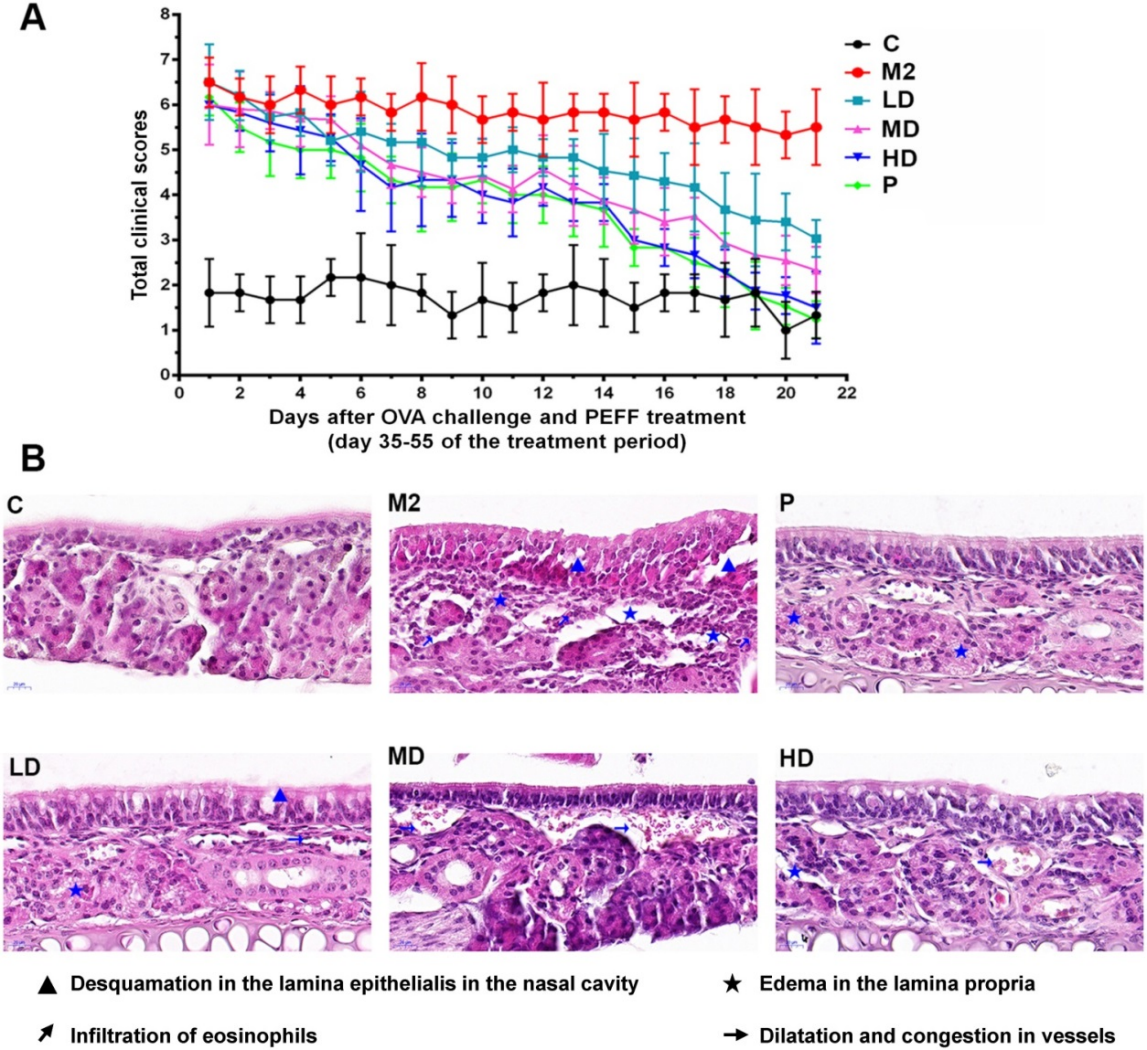
** Effects of PEFF on nasal symptoms and nasal mucosa histology in the AR mouse model.** (**A**) Symptom scores. (**B**) Representative H&E-stained sections of the nasal mucosa (×400). C, control; M2, model; P, positive control (BUD); LD, low-dose PEFF; MD, medium-dose PEFF; and HD, high-dose PEFF groups. Data are presented as the mean ± SD.

On days 35 through 55, mice with AR were treated i.n. with 20% DMSO/saline (M2), low, medium, or high doses of PEFF, or BUD (positive control) once daily. Compared with the saline-treated model group, PEFF-treated mice exhibited a dose-dependent reduction in AR nasal symptom scores (Fig. 3A). PEFF treatment also improved the histological changes in the nasal tissue of mice with AR. Whereas the nasal cavity of mice in the model group exhibited typical features of AR in the mucosa and submucosa, the mice in the HD PEFF group showed markedly reduced edema and inflammatory cell infiltration, and smaller reductions were noted in the LD and MD PEFF groups. As expected, the positive control group (BUD) also displayed marked improvements in the nasal mucosa pathology compared with the model group (Fig. 3B).

### PEFF effects on serum OVA-specific IgE, IL-4, and IFN-γ levels in mice with AR

**Figure 4:**
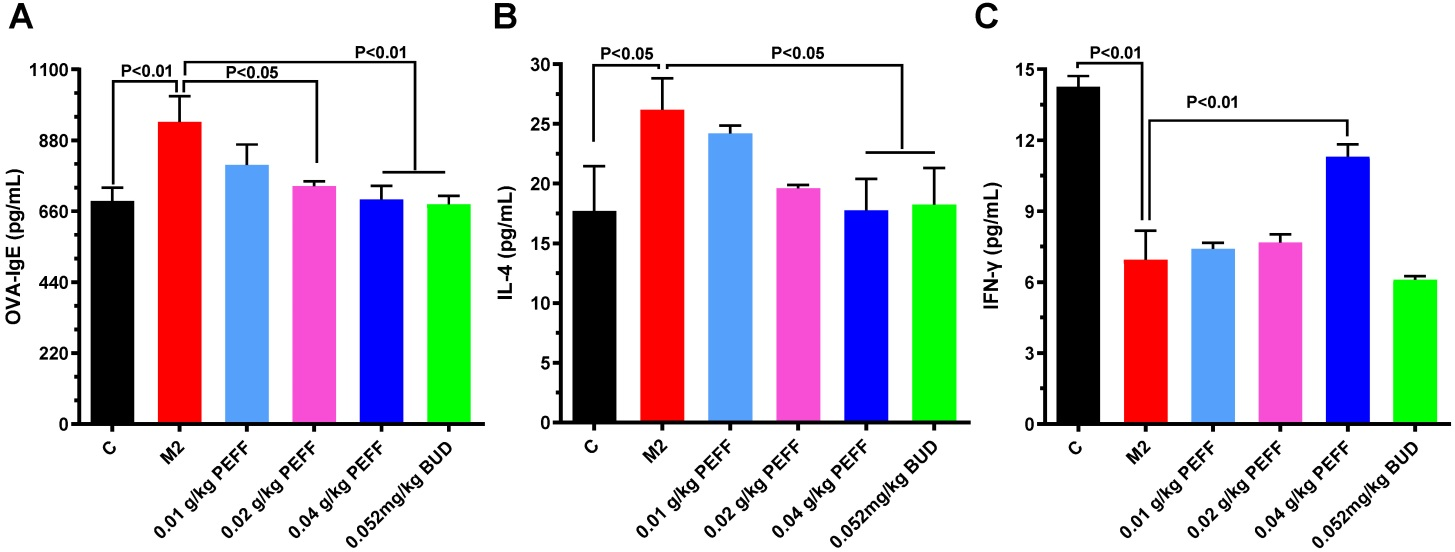
** Effects of PEFF on serum levels of OVA-specific IgE, IL-4, and IFN-γ in the AR mouse model.** (**A**-**C**) Serum levels of OVA-specific IgE (**A**), IL-4 (**B**), and IFN-γ (**C**) were measured by ELISA. Differences between groups were determined using two-tailed Student's t test. Data are presented as the mean ± SD. C, control; M2, model.

To determine whether PEFF could regulate the production of allergic mediators, we measured serum levels of IL-4, IFN-γ, and OVA-specific IgE by ELISA. In comparison with the control mice, the model group had significantly higher serum levels of OVA-specific IgE and IL-4 (Fig. 4A and B, P < 0.05) but significantly lower levels of IFN-γ (Fig. 4C, P < 0.05). Notably, treatment with HD PEFF (0.04 g/kg) or BUD significantly reduced OVA-specific IgE and IL-4 serum levels and concomitantly increased IFN-γ levels (P < 0.05, Fig. 4).

### PEFF effects on the expression of GATA3 and T-bet and the imbalance between Th1 and Th2 cells in mice with AR

**Figure 5:**
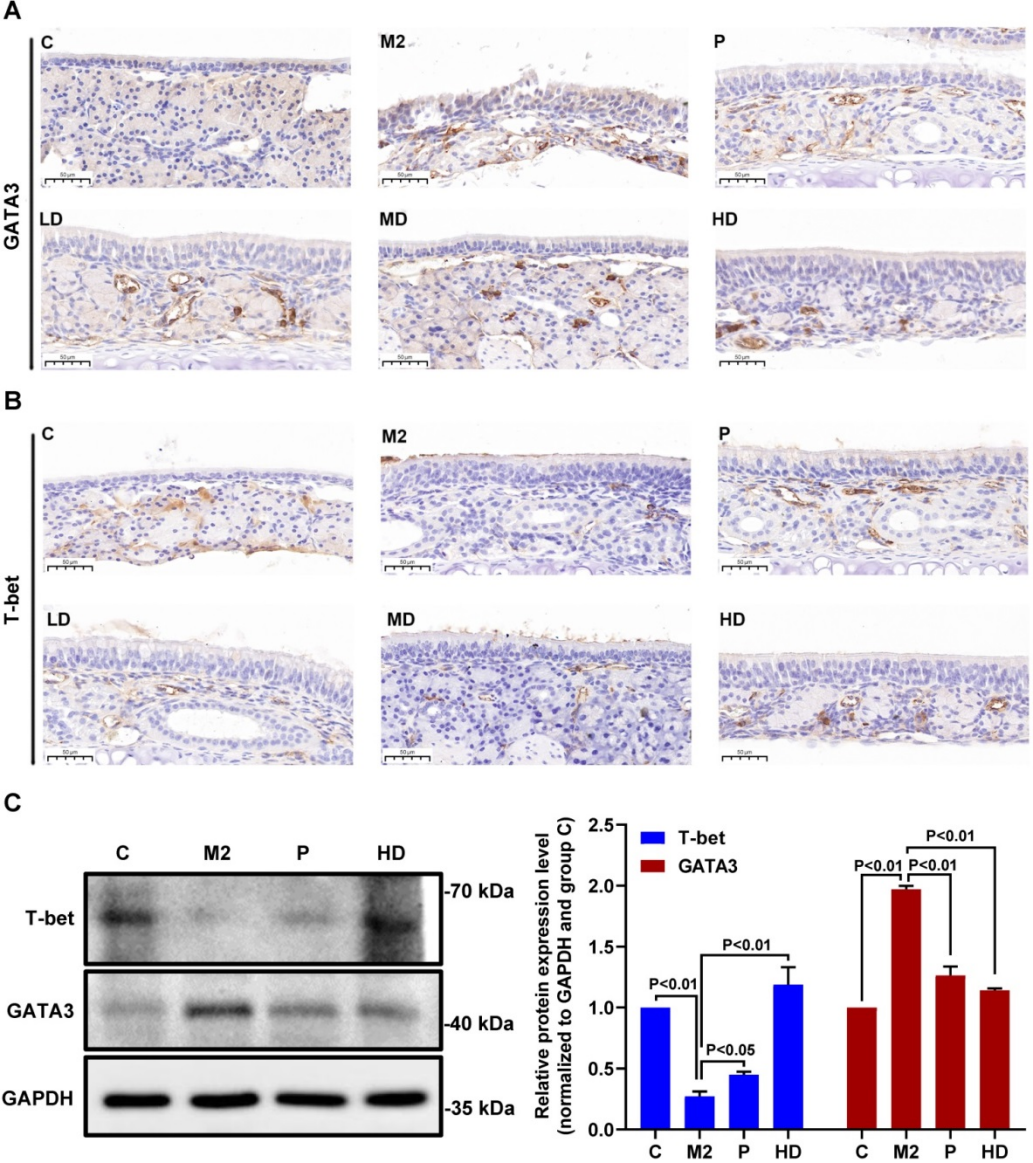
** Effects of PEFF on T-bet and GATA3 protein expression in nasal mucosa and spleen tissues of mice with AR.** (**A**, **B**) Representative images of GATA3 (**A**) and T-bet (**B**) protein expression in nasal mucosa tissue (×400). (**C**) Western blot analysis of T-bet and GATA3 protein in spleen tissue. **C,** Control; M2, model; P, positive control (BUD); LD, low-dose PEFF; MD, medium-dose PEFF; and HD, high-dose PEFF groups. Differences between groups were determined using two-tailed Student's t test. Data are presented as the mean ± SD.

Next, we investigated the molecular mechanisms by which PEFF might alleviate AR. The transcription factors T-bet and GATA3 are crucial regulators of the differentiation of precursor cells into Th1 cells, which produce IFN-γ, and Th2 cells, which produce IL-4. Therefore, we examined the effects of PEFF treatment on T-bet and GATA3 protein expression in the nasal mucosa of mice with AR. IHC staining of the proteins was quantified optically using image analysis and the results are presented as the MOD value. Compared with the control group, the nasal mucosa of mice in the model group showed increased expression of GATA3 (Fig. 5A, Table 2; P < 0.01) and decreased expression of T-bet (Fig. 5B, Table 2; P < 0.01). Consistent with its effects on serum IL-4 and IFN-γ levels, PEFF treatment increased T-bet expression and decreased GATA3 expression compared with the model group (Fig. 5A and B). These differences were significant in all treatment groups except the low-dose PEFF group (P < 0.05, Table 2).

Because the spleen is the largest peripheral immune organ, contains large numbers of lymphocytes and macrophages, and is central to the innate and adaptive immune responses 24, we also examined T-bet and GATA3 protein expression in spleen tissues by western blot analysis. We found that GATA3 levels were increased and T-bet levels were decreased in the spleens of the AR model mice compared with the control group. Moreover, administration of PEFF decreased GATA3 expression and increased T-bet expression compared with the spleens of the model group (Fig. 5C), which was consistent with the protein expression pattern in nasal mucosa.

These findings clearly demonstrate that PEFF treatment inversely affects T‑bet and GATA3 protein expression and IL-4 and IFN-γ production, suggesting that the therapeutic efficacy of PEFF in AR is mediated through modulation of Th1/Th2 responses.

## Discussion

The early-phase reaction of AR begins within a few minutes after the sensitized individual is exposed to the allergen 25. Allergen binding to IgE on mast cells and basophils induces rapid degranulation and release of preformed mediators such as histamine 26. These events cause the acute signs and symptoms of allergy: sneezing, runny nose, and itching 4, 27. At the late-phase reaction of AR, eosinophils release eosinophil cationic protein, eotaxin, and proinflammatory mediators that cause mucosal damage and edema 28, 29. In the present study, OVA sensitization followed by i.n. challenge induced a typical AR response; that is, nasal symptoms such as sneezing, nasal scratching, and watery rhinorrhea, alterations in the nasal mucosa architecture accompanied by eosinophil infiltration; and changes in serum levels of antigen-specific IgE, IL-4, and IFN-γ. These results indicate that AR was successfully modeled in BALB/c mice.

Tussilagone (TSL) is an anti-inflammatory sesquiterpene compound which separated by petroleum ether from *Farfarae Flos* 30, 31. Most recently, Jin et al. reported that TSL suppresses allergic responses in OVA-induced allergic rhinitis guinea pigs 32, indicating the active components extracted from *Farfarae Flos* have potential therapeutic effect on allergic rhinitis. Therefore, we evaluated the effects of PEFF on the symptoms of mice with OVA-induced AR and investigated the underlying mechanisms of action. The results showed that PEFF improved the nasal allergy symptoms of AR mice, which was accompanied by a reduction in tissue damage and inflammatory cell infiltration in the nasal mucosa, thereby maintaining the integrity of the nasal mucosa. In addition, PEFF reduced serum OVA-specific IgE and IL-4 levels and increased IFN-γ, likely by regulating the imbalance between Th1 and Th2 cell differentiation via modulation of the transcription factors GATA3 and T-bet.

The main immunopathological feature of AR is an imbalance between the differentiation of Th1 and Th2 cells, with Th2 cells predominating 19, 33. A number of intracellular signaling pathways and transcription factors have been shown to play key roles in driving directional Th cell differentiation 34. T-bet, a member of the t-box gene family; is selectively expressed in Th1 cells, where it not only promotes Th1 cell differentiation and IFN-γ secretion but also inhibits GATA3 expression and influences IL-4 signaling and Th2 cell differentiation 35. T-bet deficient mice exhibit defects in IFN-γ production and express excessive levels of Th2 cytokines 36. GATA3 is a member of the GATA transcription factor family. Previous studies have demonstrated that IL-4 induces the expression of GATA3 by activating STAT6, which further promotes IL-4 gene transcription and Th2 cell differentiation. GATA3 inhibits the development of Th1 cells and the secretion of cytokines such as IFN-γ, and it also induces T cells to become Th2 cells 37, 38. Therefore, the high GATA3 expression and low T-bet expression suggest local imbalance between Th1 and Th2 cell differentiation 39. In the present, IHC and western blot analysis showed that T-bet and GATA3 proteins were downregulated and upregulated, respectively, in the nasal mucosa and spleen tissues of AR mice compared with control mice, which would favor Th2 cell differentiation. PEFF treatment reversed this pattern of protein expression, thereby correcting the imbalance in Th1 and Th2 cell differentiation.

In conclusion, our study of the AR mouse model demonstrated that PEFF exerts a potent anti-inflammatory effect, which ameliorated the rhinitis symptoms, reduced the nasal histopathology, inhibited the release of proinflammatory mediators, and regulated the differentiation of Th1 and Th2 cells. These findings provide support for the use of PEFF and traditional Chinese medicine in the prevention and treatment of AR.

## Figures and Tables

**Table 1:** Nasal symptom scoring in the AR mouse model

Score	Nasal symtoms		
	Number of sneezes per 20 minutes	Nasal mucus	Number of nasal rubbing per minute
0	None	None	None
1	1-3	Nostril	1-2
2	4-10	Outflow nostril	3-5
3	≥11	Flow to face	≥6

**Table 2:** Quantification of T-bet and GATA3 protein immunoreactivity in nasal mucosal tissues of mice with AR

Groups	n	T-bet	GATA3
Control group	8	0.0141±0.0034	0.0068±0.0016
M2 (model group)	8	0.0027±0.0014^##^	0.0268±0.0074^##^
Positive control group (BUD)	9	0.0134±0.0033^**^	0.0110±0.0051^**^
LD group (0.01 g/kg PEFF)	9	0.0045±0.0028	0.0200±0.0027
MD group (0.02 g/kg PEFF)	9	0.0068±0.0033^*^	0.0132±0.0034^**^
HD group (0.04 g/kg PEFF)	9	0.0133±0.0022^**^	0.0119±0.0037^**^

Data are presented as the mean optical density per μm^2^ ± SD of five fields per section. Differences between groups were determined using two-tailed Student's t test. ^##^P<0.01 vs the Control group; *P<0.05 and **P<0.01 vs the M2 group.

## Abbreviations

PEFF: Petroleum extract of Farfarae Flos
AR: Allergic rhinitis
OVA: Ovalbumin
PBS: Phosphate buffer solution
H&E: Hematoxylin and eosin
BUD: Budesonide
DMSO: Dimethyl sulfoxide
ELISA: Enzyme-linked immunosorbent assay
Thl: Type 1 helper T cell
Th2: Type 2 helper T cell
T-bet: T-box expressed in T cells
GATA3: GATA-binding protein 3
IFN-γ: Interferon-γ
IL-4: Interleukin-4
IHC: Immunohistochemistry
